# The effect of the colour red in 20 years of Olympic taekwondo

**DOI:** 10.1038/s41598-023-49103-3

**Published:** 2023-12-08

**Authors:** Gennaro Apollaro, Coral Falcó, Verónica Morales-Sánchez, Antonio Hernández-Mendo

**Affiliations:** 1 Department of Neuroscience, Rehabilitation, Ophthalmology, Genetics and Maternal Child Health, University of Genoa, Genoa, Italy; 2 Department of Sport, Food and Natural Sciences, Western Norway University of Applied Sciences, Bergen, Norway; 3 Department of Social Psychology, Social Anthropology, Social Work and Social Services, University of Málaga, Malaga, Spain

**Keywords:** Psychology, Human behaviour

## Abstract

The objectives of this study were (1) to analyse the relationship between the colour of the protectors and the outcome of 895 matches in the six Olympic Games in which taekwondo has been included, and (2) to analyse the effect of confounding factors through the different degrees of asymmetry between contestants. Data were recorded on the colour of each athlete’s protectors, the scoring system, the sex of the athlete, the weight category, the round of competition, the winner of the match, the method of victory and the points scored by each athlete. Among the main results, a relationship emerged between male athletes wearing red and winning the match (*p* = 0.034) under the manual scoring system. There were relationships between female athletes wearing blue and winning the match in the quarterfinal (*p* = 0.014) and last 16 rounds (*p* = 0.021) using the manual and electronic scoring systems respectively. In female athletes, relationships emerged between wearing blue and winning the match with small (*p* = 0.008) and medium (*p* = 0.047) asymmetry under the manual system and with large (*p* = 0.036) asymmetry under the electronic system. The electronic system had a positive impact on the fairness of Olympic competition. Red tends to gain in importance as the asymmetry between the two athletes decreases, but not enough to give a competitive advantage. The results for Olympic competition held in the now concluded era of the manual system confirmed the presence of the colour effect as a result of psychological factors attributable to referees and judges.

## Introduction

In a brief communication published in *Nature* in 2005, evolutionary anthropologists R. A. Hill and R. A. Barton tested for the first time whether wearing red could influence the outcome of male competitions in the four combat sports (boxing, taekwondo, Greco-Roman wrestling and freestyle wrestling) that traditionally use red and blue to visually differentiate the contestants^1^. The results showed that wearing red sportswear during the 2004 Olympic Games (OG) in Athens had a positive and significant impact on the outcome of the match. Furthermore, considering the role of other factors (such as skill and strength), the authors found that only in matches between athletes of similar ability were there significantly more red winners. The hypothesis formulated by the two authors is that red improves performance through psychological effects (evolutionary and cultural associations of red with dominance and aggressiveness) on the wearer and/or the opponent^1^. Right from the start, this pioneering study gave rise to a heated debate, and almost twenty years after its publication it has generated an important body of research that is still extremely significant^2^. A few months later, Rowe et al.^3^ tested the validity of the hypotheses formulated by Hill and Barton^1^ by analysing another Olympic combat sport, judo, which uses white instead of red. They found a significant winning tendency in the 2004 OG for the athletes in blue. Thus, the authors downgraded the special effect of red and hypothesised a perceptual rather than psychological effect, related to brightness, which gives athletes in blue a visual advantage in anticipating the moves of opponents in white. In an immediate response, Barton and Hill^4^ found no colour effect in female competition in the same sports in which significant effects were found for male contestants (judo, taekwondo and freestyle wrestling) at the 2004 OG. On this occasion, the authors substantiated their initial hypothesis that the impact of colour acts through its psychological and hormonal influences and that sexual selection may have influenced the evolution of the human response to colour. Therefore, the hypothesis formulated by Rowe et al.^3^ was considered unlikely, as it was not supported by the results from the sex analysis. Furthermore, the advantage of blue was subsequently related to the fact that darker shades are seen as more dominant than lighter ones^5^.

A few years later, Hagemann et al.^6^ hypothesised that the perception of colours triggers a psychological effect in referees, rather than in the two athletes fighting, which may lead to athletes in red being awarded more points than those in blue. The authors conducted an experiment in which the same male and female taekwondo referees watched fights of male athletes of similar ability twice, but with the colours digitally reversed. The athletes in red received more points even when their performance was identical. This not only confirmed the hypothesis, but also made it possible to explain why the colour effect is stronger when athletes have similar abilities^1^. Hagemann et al.^6^ thus demonstrated that although the psychological and perceptual effects hypothesised above^1,3^ may exert an influence on performance, the referees are primarily responsible for the advantage passed on to the athletes in red. In this context, the authors suggested that for future competitions to be fairer and more transparent, technological assistance to referees in scoring would be necessary^6^. 15 years after this suggestion, it can be said that taekwondo is the Olympic combat sport that has made the greatest technological advances^7–10^. The gradual implementation of the electronic point recording system (which currently limits the “task” of referees to punches and penalties^11^) and the instant video replay system began after the Beijing 2008 OG and their impact on match fairness has been systematically monitored over time. The evidence accumulated in the electronic era^7–10^ seems to delineate a fair competitive framework from the national to the international level, in which it is not possible to speak of a colour effect, confirming the hypothesis of Hagemann et al.^6^ and the usefulness of their suggestion. However, if referees were responsible for the positive and significant impact of the colour red on match victory in the manual era^6–10^, why did Barton and Hill^4^ previously find no colour effect in the female taekwondo competition at the 2004 OG?

Firstly, Hagemann et al.^6^ documented a psychological effect in male and female referees who judged male taekwondo combats, but no study has replicated the experiment on female combats. In addition, the authors^6^ attributed a minimal role to the psychological influences of red related to the two interacting subjects^1^ but did not exclude them, and the literature accumulated over the years has confirmed this^5,12,13^. Thus, it seems safe to hypothesise that psychological effects may also have been present, and particularly specific, among the male and female referees who judged taekwondo matches with male and female athletes in the manual era. In this connection, the sex of the referees in the 2004 OG was never taken into account, although it may have contributed to the differences in the colour effect between male and female athletes. Secondly, research on the effect of colour in taekwondo has focused more on electronic era competitions, probably because analysing a scoring system that is no longer used may not be useful in practice^10^. Therefore, the fairness found in the female taekwondo competition at the 2004 OG may represent an isolated case, as we have no other data for female competitions in the manual era. In support of the importance of constantly monitoring the phenomenon and analysing it as a whole for greater understanding^2,9^, Seife^14^ found a situation of equity in the male taekwondo competition at the 2008 OG, also questioning the validity of the assumptions previously made for the male competition^1^.

In this context, the overall analysis of all male and female Olympic taekwondo competitions could help us understand the direction and magnitude of the effect of colour in the now concluded manual era and potentially confirm the situation of equity found in the current electronic era. Therefore, the main objectives of this study are (1) to analyse the relationship between the colour of the protectors and the outcome of the match in the six OGs in which taekwondo has been included; (2) to analyse the effect of confounding factors, such as differences in skill and strength between athletes, by analysing the different degrees of asymmetry. First, we hypothesise that in the manual era the effect of the colour red was significantly present in male Olympic competition. At the same time, we hypothesise that the direction and magnitude of the colour effect in female competition could be interpreted by analysing the sex of the referees. Secondly, we do not expect any effect of colour in the electronic era for either male or female Olympic competition. Finally, the analysis of different degrees of asymmetry in the electronic era should help us understand the direction and magnitude of the colour effect related to the two interacting athletes.

## Methods

### Participants

This study included all 895 matches from the six OGs in which taekwondo has been included. In line with Hill and Barton^1^, all bouts that ended as withdrawn or won by disqualification were excluded. The sample, thus organised, comprised 878 matches (male = 442; female = 436): Sydney 2000 = 127, Athens 2004 = 149, Beijing 2008 = 148, London 2012 = 151, Rio 2016 = 149 and Tokyo 2020 = 154. In the six Olympic competitions, 164 referees (male = 115; female = 49) took turns refereeing and judging matches. In line with Hill and Barton^1^ and Rowe et al.^3^, data were taken from the Official Results Book of each Games available on the open-source website [https://library.olympics.com/Default/accueil.aspx (accessed on 24 July 2022)]. No approval by an ethics committee was required for the study, according to the rules laid down by the Belmont Report^15^, since it did not involve any kind of human experimentation. Informed consent from subjects was not necessary because the measures listed in the following paragraphs were obtained in secondary form (made public by the body regulating the competition) and not from direct experimentation. The study was conducted in accordance with the Declaration of Helsinki^16^, ensuring the anonymity and privacy of the subjects.

### Measures

#### Matches

Data were recorded on the colour (blue or red) of each athlete’s protectors, the scoring system (manual: Sydney 2000, Athens 2004, Beijing 2008; electronic: London 2012, Rio 2016, Tokyo 2020), the sex of the athlete (male or female), the weight category (fly, feather, middle, heavy), the round of competition (last 16, quarterfinal, semifinal, final, repechage, bronze medal), the winner of the match, the method of victory (won by points/final score, won by points gap, won by sudden death point/ golden point, won by superiority, won by referee stops contest/knockout, withdrawn, won by disqualification, won by referee’s punitive declaration) and the points scored by each athlete. The different degrees of relative ability were also taken into consideration.

#### Referees

Data were recorded on the sex of the referees (male or female), the scoring system (manual: Sydney 2000, Athens 2004, Beijing 2008; electronic: London 2012, Rio 2016, Tokyo 2020) and the referee composition of the match (1 central referee and 3 or 4 judges).

### Statistical analysis

Chi-square (χ^2^) tests were performed to identify the associations between the colour of the protectors and the outcome of the match. Effect size (ES) was reported, using Cramér’s V, as weak (0.05–0.09), moderate (0.10–0.14), strong (0.15–0.24) or very strong (> 0.25)^17^. When the initial χ^2^ test of association was found to be statistically significant, odds ratios (OR) and 95% confidence intervals (95% CI) were calculated^18^. In line with Hill and Barton^1^, matches were recoded into different classes of asymmetry on the basis of the difference in points scored by each athlete. Each Olympic Games was categorised on the basis of the quartile of the final points difference in the bout, where the first quartile represents symmetrical contests between athletes of similar ability and the fourth quartile represents contests between athletes with large asymmetries in ability. Matches stopped early (won by referee stops contest/knockout) were scored as highly asymmetric contests and coded in the fourth quartile. χ^2^ goodness-of-fit tests were performed to compare the observed distributions of the sex and composition of the match referees with the expected distributions. Also, for these analyses, ES was reported using Cramér’s V. Statistical significance was accepted at *p* < 0.05. Data were analysed using IBM SPSS Statistics for Windows, version 25.0 (IBM Co., Armonk, NY, USA).

## Results

### Matches

In the male sample, a moderate relationship emerged between athletes wearing red protectors and winning the match under the manual scoring system (χ^2^ = 4.48, *p* = 0.034; V = 0.10; OR = 0.66, 95% CI = 0.46–0.97).

Specifically, the results showed that there were strong relationships between male athletes wearing red protectors and winning the match in the middleweight (χ^2^ = 5.14, *p* = 0.023; V = 0.21; OR = 0.42, 95% CI = 0.20–0.89) and heavyweight (χ^2^ = 5.54, *p* = 0.019; V = 0.23; OR = 0.39, 95% CI = 0.18–0.86) categories using the manual scoring system.

**Figure 1:**
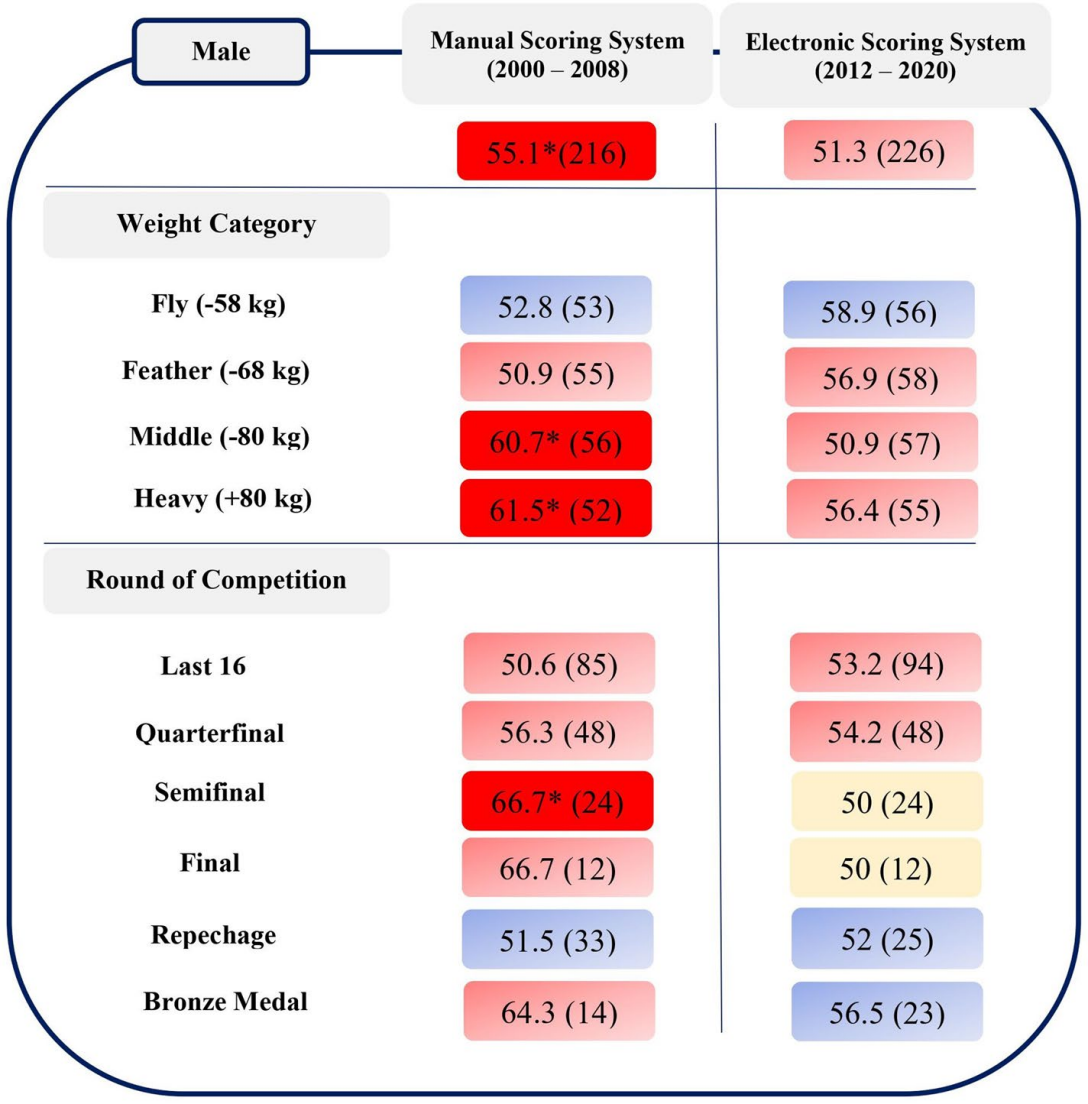
Percentage (%) of matches won by male athletes with their predominant colour protectors in each scoring system, by weight category and round of competition, showing significant association (*p < 0.05) between colour of protectors and winning the match. The figure in parentheses indicates the number of matches.

Furthermore, the results showed that there was a very strong relationship between male athletes wearing red protectors and winning the match in the semifinal round (χ^2^ = 5.33, *p* = 0.021; V = 0.33; OR = 0.25, 95% CI = 0.075–0.83) under the manual scoring system (Fig. 1).

**Figure 2:**
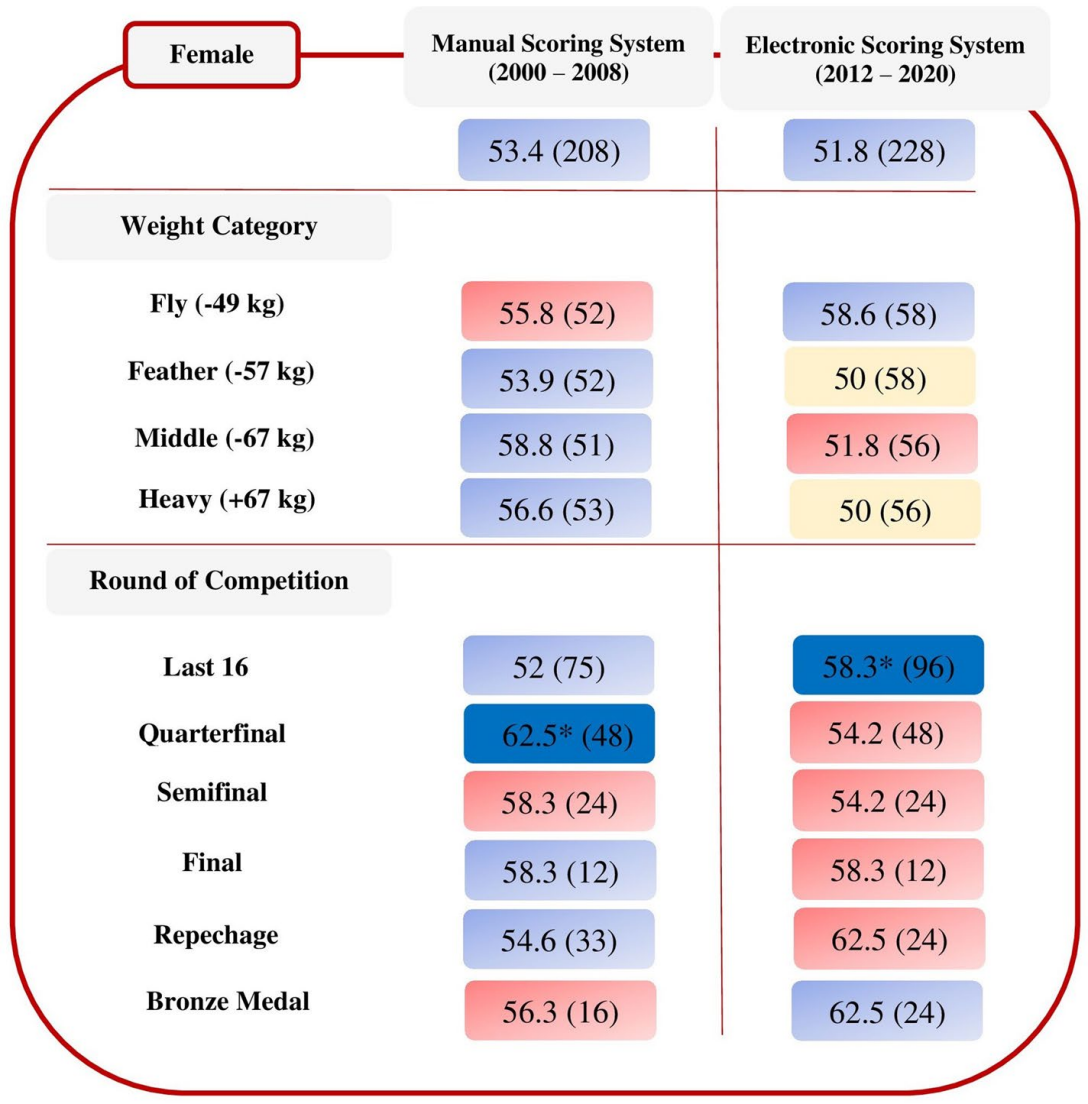
Percentage (%) of matches won by female athletes with their predominant colour protectors in each scoring system, by weight category and round of competition, showing significant association (*p < 0.05) between colour of protectors and winning the match. The figure in parentheses indicates the number of matches.

For females there was a very strong relationship between athletes wearing blue protectors and winning the match in the quarterfinal round (χ^2^ = 6.00, *p* = 0.014; V = 0.25; OR = 2.78, 95% CI = 1.22–6.35) using the manual scoring system and a strong relationship between athletes wearing blue protectors and winning the match in the last 16 round (χ^2^ = 5.33, *p* = 0.021; V = 0.17; OR = 1.96, 95% CI = 1.10–3.48) using the electronic scoring system (Fig. 2).

The relationship between the colour of the protectors and the outcome of the match was analysed for each degree of asymmetry by scoring system and sex.

**Figure 3:**
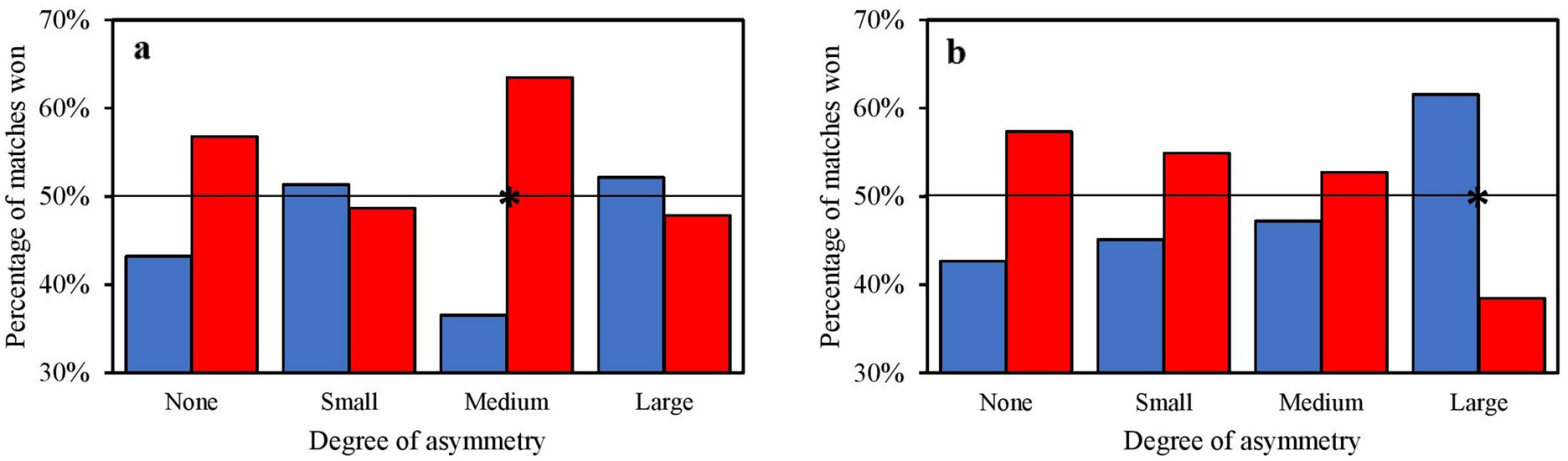
Percentage (%) of matches won by male athletes according to their colour protectors by different degrees of relative ability (asymmetry) between the two athletes in each match. (**a**) Era of manual scoring system. (**b**) Era of electronic scoring system. * = Significant association between the colour of protectors and the outcome of the match. Black lines at 50% indicate the expected percentage of wins by red or blue under the null hypothesis that colour has no effect on contest outcomes.

In male contestants, the results showed a very strong relationship between wearing red protectors and winning the match with medium asymmetry (χ^2^ = 7.54, *p* = 0.006; V = 0.27; OR = 0.33, 95% CI = 0.15–0.74) using the manual scoring system and a strong relationship between wearing blue protectors and winning the match with large asymmetry (χ^2^ = 5.54, *p* = 0.019; V = 0.23; OR = 2.56, 95% CI = 1.16–5.64) using the electronic scoring system (Fig. 3).

**Figure 4:**
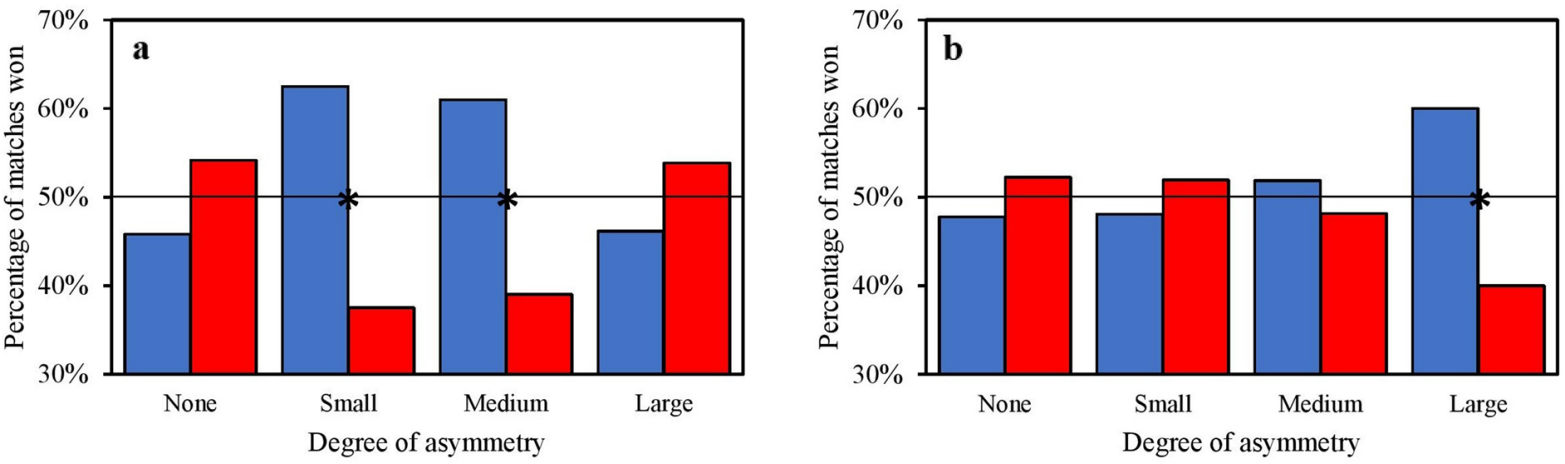
Percentage (%) of matches won by female athletes according to their colour protectors by different degrees of relative ability (asymmetry) between the two athletes in each match. (**a**) Era of manual scoring system. (**b**) Era of electronic scoring system. * = Significant association between the colour of protectors and the outcome of the match. Black lines at 50% indicate the expected percentage of wins by red or blue under the null hypothesis that colour has no effect on contest outcomes.

In female athletes, the results showed a very strong relationship between wearing blue protectors and winning the match with small asymmetry (χ^2^ = 7.00, *p* = 0.008; V = 0.25; OR = 2.78, 95% CI = 1.29–5.97) and a strong relationship between wearing blue protectors and winning the match with medium asymmetry (χ^2^ = 3.95, *p* = 0.047; V = 0.22; OR = 2.44, 95% CI = 1.00–5.93) using the manual scoring system. There was also a strong relationship between wearing blue protectors and winning the match with large asymmetry (χ^2^ = 4.40, *p* = 0.036; V = 0.20; OR = 2.25, 95% CI = 1.05–4.83) using the electronic scoring system (Fig. 4).

### Referees

The observed sex distribution of the referees under the manual scoring system was significantly skewed (χ^2^ = 33.78, *p* = 0.0001; V = 0.66), with an over-representation of male referees (83.1%). As a result, the referee composition of matches was also significantly skewed (χ^2^ = 270.85, *p* = 0.0001; V = 0.95), with an over-representation of matches in which more than half the referees were male (97.4%).

In contrast, the observed sex distribution of the referees under the electronic scoring system was equally distributed (χ^2^ = 2.59, *p* = 0.11; V = 0.17). Therefore, the referee composition of matches was also equally distributed (χ^2^ = 1.73, *p* = 0.19; V = 0.06), with a percentage of matches in which male referees made up more than half of the referee composition similar to that in which female referees made up half or more of the referee composition.

## Discussion

The study of the relationship between the colour of the protectors and the outcome of the match in the male Olympic taekwondo competition held in the now concluded era of the manual scoring system revealed a significant effect of the colour red. In particular, wearing red had a positive impact on winning the match in the heaviest weight categories and in the semifinal round. In contrast, the study of this phenomenon in Olympic competition under the current electronic scoring system revealed no colour effect (Fig. 1). In support of the importance of constantly monitoring the phenomenon and analysing it as a whole for greater understanding^2,9^, this is the first study that has investigated the effect of colour in taekwondo by monitoring the same competition over a twenty-year period, allowing a direct comparison between the two different scoring systems. In this respect, the results of comparing the two eras corroborate the hypothesis of Hagemman et al. and the usefulness of their suggestion^6^. Although the literature has verified that psychological and perceptual effects related to interacting subjects^1,3^ can exert an influence on performance^5,12,13^, it is more likely that referees were primarily responsible for the advantage conferred on athletes in red in the manual era. The implementation of the electronic scoring system has had a positive impact on the fairness and objectivity of the match^7–10^, by establishing a competitive framework in which it is not possible to speak of a colour effect.

In the female Olympic taekwondo competitions held in the manual era, no colour effect emerged. However, wearing blue positively influenced the outcome of the match in the quarterfinal round. Similarly, no colour effect emerged in Olympic competition in the electronic era, although wearing blue had a positive impact on winning the match in the last 16 round (Fig. 2). Although on the one hand the equality found in the electronic era is in line with what was found in male competition and in the more recent literature^7–10^, on the other, the situation of equity found in the manual era extends what was found by Barton and Hill^4^ in the 2004 OG. The latter point raises doubts about the validity of the hypotheses of Hagemann et al.^6^ and consequently of our hypothesis formulated for male competition. Indeed, if in the manual era referees were responsible for the positive and significant impact of the colour red on match victory, why did this bias affect only male competition? Is it therefore more likely that Hill and Barton’s initial hypothesis^1^ (i.e., that psychological and hormonal factors and sexual selection may have influenced the evolution of the human response to colours) can explain what emerged in the manual era?

Within this debate and these opposing hypotheses, one possible explanation could lie in the middle. The documented psychological effects^5,12,13^ may also have been present, and particularly specific, among male and female referees and judges who assessed the matches of male and female athletes in the manual era. However, the sex of the referees has never been taken into account, despite the fact that it may have contributed to the differences in the effect of colour between male and female athletes in the manual era. Exploring this issue for the first time in our study, we found an over-representation of male referees and judges among those assessing Olympic competition in the manual era. Consequently, in almost all the matches contested, more that half the referees were male. First, it is interesting to note that Hagemann et al.^6^ documented a psychological effect in male and female referees who judged male taekwondo bouts, but no study has replicated the experiment in female bouts. It therefore seems inappropriate to assume a priori that the effect was same, or that there was no effect, in the reverse situation. Secondly, Little and Hill^5^, studying perceptions of aggression, dominance and physical competitiveness, hypothesised on the basis of their findings that dominance might be more salient for females judging colour when using it to assess male quality, whereas males might be more sensitive to red as dominant when judging potential physical conflicts with other males. Thus, it seems safe to hypothesise that the red colour effect that emerged in male Olympic competition and the different direction and magnitude of the colour effect in female competition may reflect more specific psychological effects related to male referees and judges. In contrast, a situation of equity between male and female referees has characterised Olympic competition in the electronic age. Although this information is less significant in practice, as the “task” of referees has been gradually reduced (it is now limited to punches and warnings only^11^), it provides insights into the course of female participation in this sport. Even more, it highlights the fact that the imbalance in the manual era cannot be overlooked when interpreting the results, as it may have contributed to the differences in the effect of colour between male and female athletes.

The analysis of different degrees of asymmetry in the manual era revealed the presence of the red colour effect in matches with medium asymmetry in the male Olympic competition (Fig. 3A). In contrast, wearing blue had a positive impact on victory in matches with medium and small asymmetry in female competition (Fig. 4A). In the electronic era, this analysis revealed the presence of the blue colour effect in matches with large asymmetry, in both male and female Olympic competition (Figs. 3B and 4B). Firstly, it seems clear that Hill and Barton’s hypothesis^1^ cannot be used to explain the results of different degrees of asymmetry in the manual era, as the free influence of referees does not allow the results to be attributed more to specific psychological effects between the two interacting athletes. Secondly, our results in the manual era show that the bias of referees is not decisive when athletes are of similar abilities^6^. In particular, these results provide further insights into the specific psychological effects that may have influenced male referees in judging the matches of male and female athletes. Whereas the effect of the colour red seems to be confirmed in male Olympic competition, the different direction and magnitude of the colour effect in female competition would seem to tend towards the colour blue. Finally, the results of the analysis of the different degrees of asymmetry in the electronic era show that the direction and magnitude of the effect of colour are attributable more to specific psychological effects between the two athletes fighting. The results confirm the direction but not the magnitude of the colour effect found by Hill and Barton^1^, suggesting that red tends to gain in importance as the asymmetry between the two athletes decreases, but not enough to confer a significant competitive advantage in either male or female competition.

## Conclusions

The results of this study confirm that the electronic scoring system had a positive impact on the fairness and objectivity of the Olympic taekwondo competition. In the electronic era, the influence of referees was reduced, and therefore the direction and magnitude of the colour effect is more attributable to the mechanisms associated with the two athletes fighting. Red tends to gain in importance as the asymmetry between the two athletes decreases, but not enough to give a significant competitive advantage in either male or female competition. The results for the Olympic competition held in the now concluded era of the manual scoring system confirmed the presence of the colour effect as a result of psychological effects attributable to referees and judges. In particular, we believe that the different direction and magnitude of the colour effect between male and female competition is explained by specific psychological mechanisms derived from the imbalance in referee composition in these competitions. Overall, this study highlights the continuous evolution of Olympic taekwondo over the past two decades. Specifically, further possible future technological developments will allow for further study of the effect of colour and its impact in practice.

## Data Availability

The data used in the current study are available in the “Official Results Book” of each Olympic Games, [https://library.olympics.com/Default/accueil.aspx]. The datasets, generated from this source and analysed, are available from the corresponding author on reasonable request.
